# IFITM3 promotes glioblastoma stem cell-mediated angiogenesis via regulating JAK/STAT3/bFGF signaling pathway

**DOI:** 10.1038/s41419-023-06416-5

**Published:** 2024-01-13

**Authors:** Zhangsheng Xiong, Xiangdong Xu, Yuxuan Zhang, Chengcheng Ma, Chongxian Hou, Zhongsheng You, Lingling Shu, Yiquan Ke, Yang Liu

**Affiliations:** 1 Department of Neuro-oncological Surgery, Zhujiang Hospital, Southern Medical University, Guangzhou, 510060 PR China; 2 Key Laboratory of Neurosurgery in Guangdong Province, Southern Medical University, Guangzhou, 510060 PR China; 3 Department of Neurosurgery, Institute of Neuroscience, The Second Affiliated Hospital of Guangzhou Medical University, Guangzhou, 510260 PR China; 4 State Key Laboratory of Oncology in South China, Collaborative Innovation Center for Cancer Medicine, Sun Yat-sen University Cancer Center, Guangzhou, 510060 PR China; 5 Department of Hematological Oncology, Sun Yat-sen University Cancer Center, Guangzhou, 510060 China; 6 State Key Laboratory of Pharmaceutical Biotechnology, The University of Hong Kong, Guangzhou, PR China

**Keywords:** Oncogenesis, Tumour angiogenesis

## Abstract

Interferon-induced transmembrane protein 3 (IFITM3) has been previously verified to be an endosomal protein that prevents viral infection. Recent findings suggested IFITM3 as a key factor in tumor invasion and progression. To clarify the role and molecular mechanism of IFITM3 in Glioblastoma multiforme (GBM) progression, we investigated the expression of IFITM3 in glioma datasets culled from The Cancer Genome Atlas (TCGA) and Chinese Glioma Genome Atlas (CGGA). Primary GBM stem cells (GSCs) were cultured and identified in vitro. Loss-of-function and gain-of-function experiments were established by using shRNAs and lentiviral vectors targeting IFITM3. Co-culture system of GSCs and vascular endothelial cells was constructed in a Transwell chamber. Tube formation and spheroid-based angiogenesis assays were performed to determine the angiogenic capacity of endothelial cells. Results revealed that IFITM3 is elevated in GBM samples and predictive of adverse outcome. Mechanistically, GSCs-derived IFITM3 causes activation of Jak2/STAT3 signaling and leads to robust secretion of bFGF into tumor environment, which eventually results in enhanced angiogenesis. Taken together, these evidence indicated IFITM3 as an essential factor in GBM angiogenesis. Our findings provide a new insight into mechanism by which IFITM3 modulates GBM angiogenesis.

## Introduction

Glioblastoma (GBM) is the most aggressive brain tumor with extensive angiogenesis and poor clinical outcome [1]. Abnormally increased vascularization results in tumor progression and recurrence. Abundant pro-angiogenic factors in the tumor environment contributed greatly to endothelial cell (EC) proliferation. Among these factors, vascular endothelial growth factor (VEGF) is the most well-known and effective cytokine. Targeting blood vessels has now been an alternative treatment approach in GBM, especially recurrent GBM. Bevacizumab, the first anti-angiogenesis agent approved for glioma, neutralizing VEGF, prolongs progression-free survival (PFS) in GBM patient, however, showed minimal efficacy for overall survival (OS) [2]. These evidences indicate that further exploration in GBM angiogenesis mechanism is urgently needed.

A subpopulation of tumor cells, termed GBM stem cells (GSCs), were verified to be responsible for GBM progression, vascularization and recurrence [3]. GSCs serve as progenitors for self-renew and proliferation in glioma tissue [4]. There is emerging evidence indicating that cancer stem cells (CSCs) might support tumor progression by enhancing tumor angiogenesis. The proliferation of adjacent blood vessels or the migration of BM-derived stem cells to the tumor result in tumor endothelial cells [5]. There are considerable evidences that GSCs would promote pro-angiogenic factors in tumor microenvironment. For instance, glioma stem cells produce high level of stromal-derived factor 1, which eventually promotes local endothelial activity and systemic angiogenic processes involving bone marrow-derived endothelial progenitor cells (EPC) [6]. A study reported by Ping and et al. revealed that glioma cells co-expressing CD133 and CXCR4 promotes angiogenesis by producing VEGF [7]. By activating histamine H1 receptor (H1R) and Ca2^+^-NF-κB axis, GSCs-secreted histamine promotes angiogenesis and the progression of GBM [8]. Notably, several markers such as Sox2 and Nestin indicated that stem cells are increased in bevacizumab-resistant patients [9]. These results indicated glioma stem cell as a potential target in tumor angiogenesis. Understanding molecular mechanism involved in cancer stem cell-mediated angiogenesis is instrumental for anti-angiogenic therapy in glioma.

Interferon-inducible transmembrane proteins (IFITM) comprise a family of interferon-induced molecules, consisting primarily of IFITM1, IFITM2, IFITM3 and IFITM5 [10]. During endothelial cells sprouting process in vitro, IFITM proteins were induced expeditiously and were essential for lumenized vessel formation [11]. IFITM 1–3 have been implicated in viral pathogen restriction [12]. IFITM3 was firstly identified to restrict influenza A virus infection and influence the adaptive immune response [13]. Recent findings have indicated IFITM3 as a key role in a number of pathological process, particularly in neoplasms. IFITM3 in prostate cancer cells promotes tumor progression and bone metastasis by activating TGFβ pathways [14]. Latest research indicated that IFITM3 phosphorylation accounts for amplification of PI3K signaling, facilitating malignant transformation of B cells [15]. In fact, human ESCs (hESCs) express high levels of IFITM1 and their expression decreases with differentiation into hepatocyte-like cells [16]. And Fragilis 2 (encoding IFITM1 in mouse) was reported to be expressed in murine pluripotent embryonic stem cells and a vital downstream mediator of Wnt/β-catenin [17]. These evidences suggest that IFITM family might play a crucial role in stem cells, including cancer stem cells. Considering these evidences that IFITM proteins are critical for cell stemness and angiogenesis, we attempted to explore the role of IFITM3 in GSCs and glioma angiogenesis and understand the potentially underlying mechanism.

In the present study, we report a GSCs-mediated angiogenesis manner, in which IFITM3 expressed in GSCs regulates JAK/STAT3 signaling pathway that preferentially stimulates bFGF production, leading to tumor angiogenesis. Blocking IFITM3 expression in GSCs substantially suppresses angiogenesis, especially when in combination with anti-VEGF agent.

## Materials and methods

### Glioblastoma specimen and cell culture

Twenty-eight paraffin-embedded samples from human glioma patients (WHO I-IV) with corresponding clinic-pathological data were collected in the Department of Neurosurgery at Zhujiang Hospital from 2017 to 2019. Informed consent was obtained from all of patients. Both study protocol and informed consent were approved by the Ethical Committee of Zhujiang Hospital.

For GBM stem cell culture, tumor samples were collected from consenting patients diagnosed as GBM. GBM tissues were treated as previously reported [18]. Digested cells were cultured in DMEM/F12 (Gibco, USA) medium supplemented with EGF (20 ng/ml, Peprotech, USA), bFGF (20 ng/ml, Peprotech) and B27 (1:50, Gibco). GSCs were cultured and expanded in two methods, suspension culture of neuro-spheres in low-attached wells, and adherent culture on Laminin (Corning Biosciences, USA) -coated plates [19]. Low passage (2–4) GSCs were used in this study.

U87 and U251 cells were purchased from Chinese Academy of Sciences Cell Bank (Shanghai, China). Glioma cell lines were cultured in high glucose DMEM medium (Gibco) containing 10% fetal bovine serum (FBS, Gibco). Human brain micro-vessel endothelial cells (hBMECs) were purchased from Procell Life Science & Technology (Wuhan, China) and cultured in supplemented endothelial growth medium (EGM-2, Lonza, Walkersville, MD, USA).

### Reagents, shRNA and transfection

shRNA and lentiviral vector for IFITM3 were designed and constructed by Genechem (Shanghai, PR China). The shRNA sequences include IFITM3 shRNA-1 (5′-GCTTCATAGCATTCGCCTACT-3′), IFITM3 shRNA-2 (5′-CCTGTTCAACAC CCTCTTCAT-3′) and shRNA Scrambled control (5′-GATATGTGCGTACCTAGCA T-3′). Glioma cells were transfected with 50 nmol/L shRNAs by using Lipofectamine (Invitrogen, Carlsbad, CA, USA) according to the manufacturer’s instructions.

### Bio-informatical analysis

Gene expression profiles and corresponding clinical data in Glioma/GBM data were obtained from TCGA and CGGA databases. Primary glioma patients with complete survival data, mRNA sequencing data, and WHO Grade classification data were collected as including patients. Gene expression based on tumor grade and survival analysis were performed using R studio, GEPIA2, and Gliovis [20, 21]. GBM samples from TCGA of CGGA were divided into high and low expression groups according to the median IFITM3 expression level. Differentially expressed genes (DEGs) and Gene set enrichment analysis (GSEA) were examined using R packages DESeq2 and clusterProfiler to pick out the significantly enriched pathways based on Gene ontology terms. The summary information for the patients is presented in supplementary table S2.

### Cell Counting Kit-8 (CCK-8) and EdU cell proliferation assay

CCK-8 and EdU assays were performed to examine endothelial cell proliferative capacity. For CCK-8 assay, endothelial cells were seeded onto 96-well plates (Costar, Cambridge, MA, USA) and cultivated for 7 days. Viable cells were analyzed using Cell Counting Kit-8 (Dojindo, Kumamoto, Japan) according to the manufacturer’s instructions. For Edu assay, endothelial cells to be stained were added with EdU solution, followed by incubation, fixation, permeabilization and Edu staining (Abcam, USA).

### Cell invasion assay

Cell invasion assay was conducted using cell culture insert with 8-um pores in 24-well plates (Costar, USA). Insert was pre-coated with Matrigel (Corning) and the bottom chamber was filled with 0.5 mL medium containing 10% FBS. Cells (1 × 10^5^) suspended in 100 μl DMEM medium were placed at the upper chamber and incubated for 24 h. Migrated cells on the underside of the insert were stained with crystal violet.

### Tube formation assay

Tube formation assay was carried out as previously stated [22]. 96-well plate was coated with Growth factor reduced Matrigel and incubated at 37 °C for 30 min. Endothelial cells were seeded at 2 × 10^4^ cells/well and incubated for 24 h. Tube quantification was examined with ImageJ software.

### Endothelium spheroid-based sprouting angiogenesis assay

Sprouting assay was performed according to protocol reported by Korff with minor modification [23]. Dissociated endothelial cells were suspended in complete EGM-2 medium containing 0.25% methylcellulose and seeded on low attachment 96-well plates (Corning). About 800–1000 cells per well bond together within 48 h to form a single spheroid, which were then transferred to 24-well plate and cultivated in Collagen I solution (Enzo Life Sciences, USA) supplemented with 20% FBS. Sprouting capacity was measured via counting the total sprout number using NeuronJ plugin of ImageJ software.

### Immunoblotting and immunological analysis

The immunoblotting assay was performed as previously described [24]. Human ELISA kit (eBioscience) was used to measure the concentration of bFGF from culture medium according to manufacturer’s protocol. Cells were cultured in 6-well plates for 72 h and supernatants were collected for further analysis. Experiments were performed in triplicate. Antibodies used in the present study were listed in the supplementary table S3.

### Tissue Immunohistochemical (IHC) and Immunofluorescence staining (IF)

Specimens sections of surgical GBM tissues and intracranial mice xenografts were prepared in a routine procedure [25] and immuno-stained with targeted proteins using a protocol as previously described [26].

### In vivo xenograft assay

BALB/C nude mice in the present research were procured from Experimental Animal Center of Southern Medical University. Orthotopic xenograft model was established using Balb/c nude mice (6-week-old) with GBM cells (1 × 10^5^ cells in 0.1 ml PBS) stably transfected with mCherry-LUC vector based on Ozawa’s protocol [27]. Intracranial tumor growth was examined by in-vivo imaging system (IVIS Lumina II, Caliper, USA). The protocol has been registered and approved by the Animal Care and Use Committee of Southern Medical University.

### Statistical analysis

All experiments in the study were carried out at least 3 time and analyzed using Prism 8 (GraphPad Software Inc., USA) or R software. Results were displayed as Mean ± SD. Statistical significance was determined via using Student’s *t*-test or one-way ANOVA with Bonferroni correction for multiple comparisons. *P* < 0.05 was considered statistically significant.

## Results

### IFITM3 expression is elevated in human GBM tissue

**Fig. 1:**
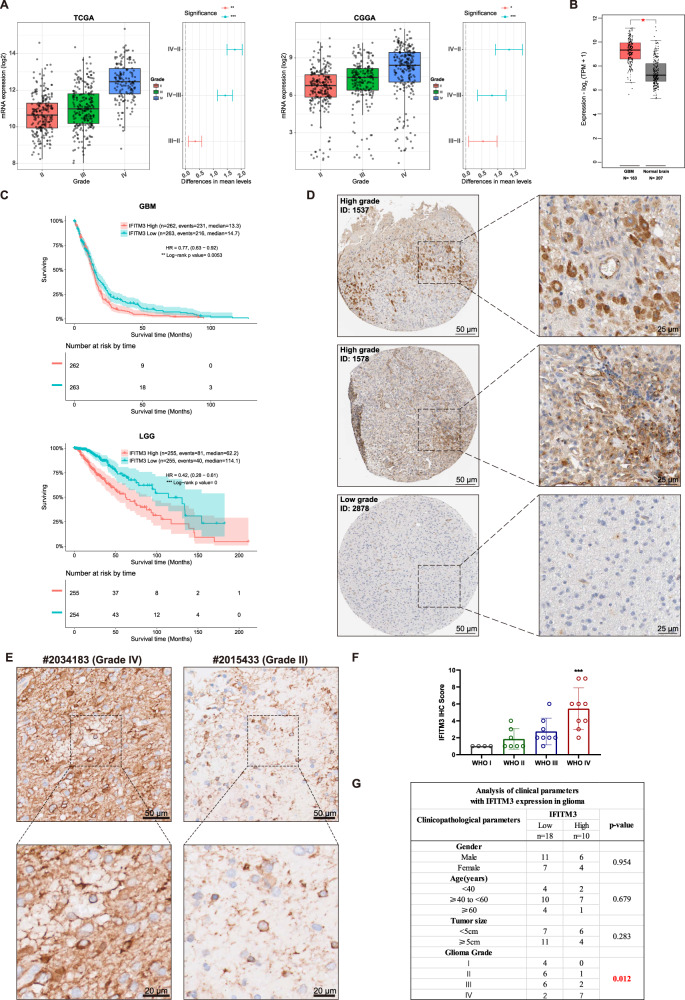
IFITM3 correlates with glioma grade and prognosis of GBM patients. **A** Gene expression profile of IGFBP4 in TCGA/CGGA glioma datasets stratified by tumor grade. **B** Gene expression analysis of IFITM3 in GBM (TCGA) and normal brain tissues (GTEx) was conducted using GEPIA2 web tool. **C** Kaplan–Meier survival analysis of GBM/LGG patients with high or low IFITM3 expression. **D** Representative images of IHC staining for IFITM3 in patients with high or low-grade glioma from Human Protein Atlas datasets. **E** Representative IHC images for IFITM3 in patients diagnosed as grade IV or Grade II glioma from Zhujiang Hospital. **F** IHC score of IFITM3 in human glioma samples stratified by tumor grade. **G** Correlation of IFITM3 expression with gender, age, tumor size, and tumor grade. Scale bar = 25/50 μm. Data are expressed as Mean ± SD. **p < 0.05, **p < 0.01, ***p < 0.001. TCGA The Cancer Genome Atlas, CGGA Chinese Glioma Genome Atlas, GBM Glioblastoma, LGG Low-grade gliomas, IHC Immunohistochemistry.

To examine the expression of IFITM3 in GBM, we investigated IFITM3 gene expression level in glioma datasets from The Cancer Genome Atlas (TCGA) and Chinese Glioma Genome Atlas (CGGA). Results showed a markedly elevated expression of IFITM3 in GBM, compared to lower grade gliomas (grade II, III) (Fig. 1A). Data from GEPIA2 web tool also revealed an obviously higher IFITM3 expression in GBM sample than that in normal brain tissue (Fig. 1B). Mesenchymal subtype of GBM has been associated with therapy resistant and more aggressive features than other subtypes [28]. Our data suggested that GBM sample with mesenchymal subtype displayed a markedly upregulated IFITM3 expression (Supplementary Fig. 1a). IFITM3 also correlated with IDH1 (Supplementary Fig. 1b) and MGMT (Supplementary Fig. 1C) status in GBM samples. And upregulated IFITM3 significantly correlated with poor outcome in both GBM and LGG samples (Fig. 1C). We examined IHC staining with IFITM3 from human protein atlas (HPA) and observed a strong intensity in high grade glioma, while a weak intensity was documented in low-grade glioma (Fig. 1D). Furthermore, we collected 28 glioma specimens with clinicopathologic data. IHC staining of glioma samples indicated a positive relation between IFITM3 expression and tumor grade (Fig. 1E, F). Moreover, patients were divided into two groups based on IFITM3 expression. Surprisingly, it revealed that IFITM3 expression was only significantly related to tumor grade, rather than gender, age and tumor size (Fig. 1G). Taken together, these data indicated that IFITM3 expression was elevated in glioma samples, especially in GBM sample.

### IFITM3 is enriched in GBM stem cells

**Fig. 2:**
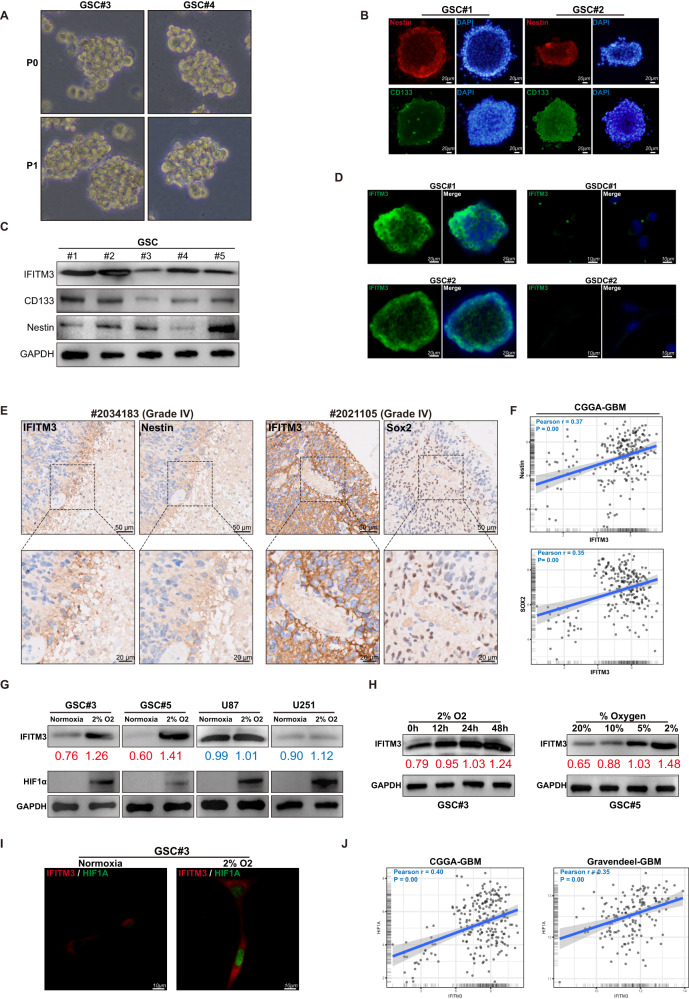
IFITM3 is enriched in glioma stem cells. **A** Sphere formation ability was identified in human GBM-derived stem cells. **B** Nestin and CD133 were identified in GSCs using immunofluorescence staining. **C** IFITM3, Nestin and CD133 expression were examined via using immunoblotting. **D** IFITM3 was detected in GSC sphere and differentiated cells (GSDCs) via immunofluorescence staining. **E** IFITM3 and molecular markers for stem cells were examined in human GBM specimens. **F** Correlation between IFITM3 expression and stem cell markers in CGGA glioma dataset was evaluated. **G** GSCs and glioma cells were cultured under normoxic or hypoxic conditions, IFITM3 and HIF1α expression were determined by immunoblotting. **H** GSCs were exposed to hypoxia at different time points or varying oxygen levels for 48 h. IFITM3 expression were analyzed using immunoblotting. **I** IFITM3 and HIF1α were examined in cells cultivated under normoxic or hypoxic environment. **J** Correlation between IFITM3 and HIF1A gene expression were assessed in CGGA and Gravendeel GBM datasets. Results are represented as Mean ± SD of biologically triplicate assays. *p < 0.05, **p < 0.01, ***p < 0.001. Scale bar = 100 μm.

Cancer stem cells (CSCs), a subpopulation inside the heterogeneous tumor tissues, are characterized by multi-lineage differentiation potential [29]. Evidences from various studies indicated that CSCs were essential for tumor progression, recurrence and metastasis [30]. In the present study, GBM stem cells (GSCs) were derived from fresh GBM samples and cultured in vitro. GSCs possessed the sphere-forming capacity (Fig. 2A) and neural stem cell markers (Fig. 2B) as reported previously. Immunoblotting confirmed that GSCs within the culture universally express IFITM3, despite some variations in levels between cells (Fig. 2C). While GSCs were enriched for IFITM3, GSC serum-differentiated cells (GSDCs) exhibited reduced expression (Fig. 2D), indicating that IFITM3 was solely expressed in neural stem cells or cancer stem cells. Moreover, we observed a positive association between IFITM3 and Nestin expression in samples from GBM patients (Fig. 2E). Likewise, in CGGA GBM dataset, IFITM3 expression closely correlated with stem cell markers Nestin and Sox2 (Fig. 2F). Sufficient evidence has established a role for hypoxia environment in stem cell maintenance [31]. Interestingly, low oxygen level induced increased IFITM3 and HIF1α expression in GSCs, rather than glioma cell lines (Fig. 2G). When GSCs were exposed to 2% oxygen, protein level of IFITM3 gradually increased over time (Fig. 2H). When exposed to varying levels of O_2_ for 24 h, GSCs exhibited rising IFITM3 expression (Fig. 2H). Similarly, GSC spheres were cultured under hypoxic environment, IFITM3 and HIF1α expression were elevated (Fig. 2I). And in CGGA and Gravendeel GBM datasets, IFITM3 was significantly correlated with HIF1α mRNA levels (Fig. 2J).

### IFITM3 in GBM stem cells regulates endothelium proliferation and sprouting

**Fig. 3:**
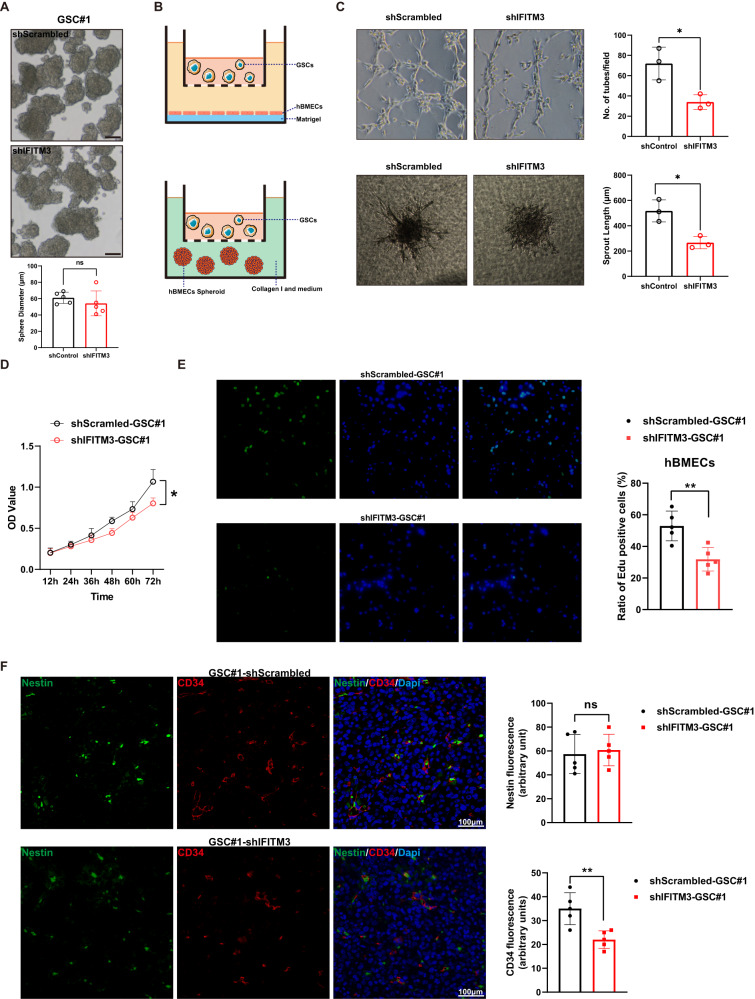
IFITM3 is linked to enhanced angiogenesis. **A** ShScrambled or shIFITM3 GSCs were cultivated in serum-free media to form spheres. Sphere diameters were measured. **B** Illustration of co-culture systems in which GSCs and hBMECs were cultivated in transwell chamber. **C** Tube formation and sprouting capacity of endothelial cells cultured with shScrambled/shIFITM3 GSCs. Bar charts show tube number and sprout length of corresponding assays. **D** Proliferation of endothelial cells cultured with shScrambled or shIFITM3 GSCs was calculated using CCK-8 assay. **E** endothelial cells cultured with shScrambled or shIFITM3 GSCs were subjected to Edu staining. **F** Intracranial tumor model was established with shScrambled or shIFITM3 GSCs. Xenograft specimen were stained with Nestin and CD34. Bar charts revealed fluorescence intensity of indicated proteins. Scale bar = 100 μm. Results are represented as Mean ± SD of biologically triplicate assays. *p < 0.05, **p < 0.01, ***p < 0.001.

To further determine the role of IFITM3 on biological function of GSCs. We examined sphere-forming competency of GSCs and found that downregulation of IFITM3 had no impact on GSC sphere-forming capacity (Fig. 3A). Since sufficient angiogenesis is essential for solid tumor expansion [32], we co-cultured GSCs with brain microvascular endothelial cells (hBMECs), and examined cell proliferation and sprouting capacity (Fig. 3B). It suggested that IFITM3 knockdown in GSCs reduced tube formation and sprouting capacity of hBMECs (Fig. 3C). Moreover, proliferative ability of endothelial cells was impaired when IFITM3 was downregulated (Fig. 3D, E). We established intracranial xenografts in nude mice through implanting GBM stem cells to evaluate the effect of IFITM3 on angiogenesis in vivo. Results indicated that IFITM3 downregulation led to reduced vessel density as assessed by CD34, while exhibited no effect on stem cell population (Fig. 3F). Together, these findings suggested that IFITM3 in GBM stem cells would be responsible for increased angiogenesis in tumor microenvironment.

### IFITM3 induces JAK/STAT3 activation in GSCs

**Fig. 4:**
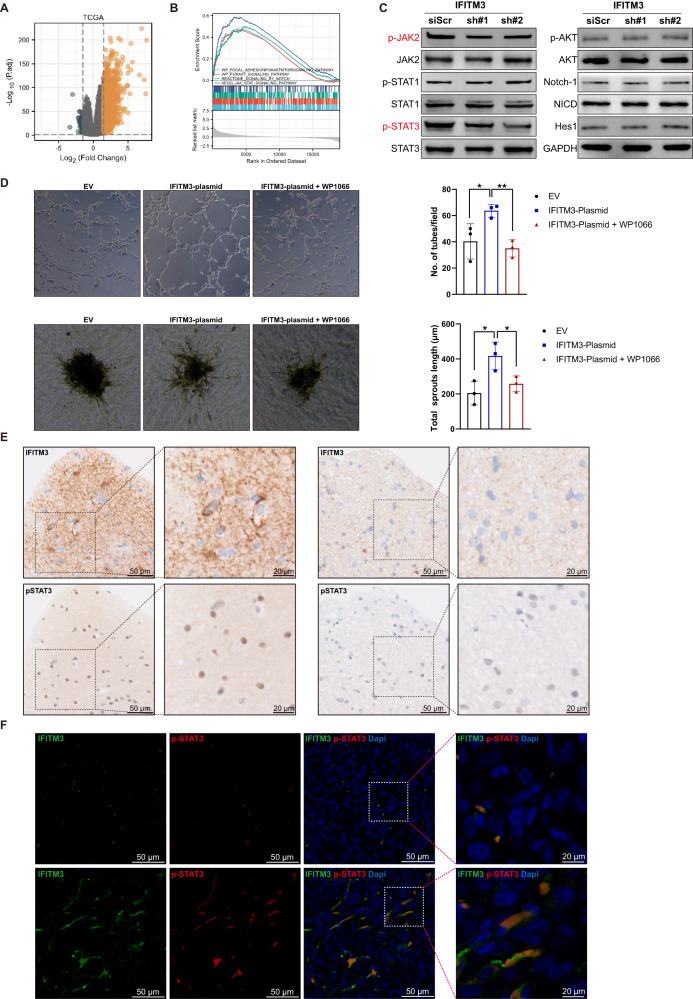
IFITM3 induces invasiveness of GBM cells via upregulating JAK/STAT signaling. **A** Volcano plot of gene expression profiles of human glioma cases from TCGA database. **B** Gene set enrichment analysis (GSEA) between high and low IFITM3 expression show enriched pathways associated with angiogenesis. **C** GSCs were treated with scrambled or shRNA targeting IFITM3, indicated proteins of JAK/STAT, PI3K/Akt, and Notch pathways were determined by immunoblotting. **D** IFITM3-expressing GSCs with/without JAK/STAT inhibitor WP1066 were co-cultured with endothelial cells, followed by Matrigel-based tube formation assay (upper panel) or sphere-based sprouting assay (lower panel) to evaluate in vitro angiogenesis capacity of the latter. Bar charts in the right panel show statistical results **E** Representative IHC images of IFITM3 and p-STAT3 from human GBM samples. **F** Representative IF images of IFITM3 and p-STAT3 from intracranial xenograft specimens. Results are represented as Mean ± SD of biologically triplicate assays. *p < 0.05, **p < 0.01, ***p < 0.001. ns not significant.

GBM patients from TCGA dataset were categorized into high and low expression groups by using median expression value of IFITM3 as cut-off point. Differentially gene expression with RNA-seq data was analyzed (Fig. 4A). Gene set enrichment analysis (GSEA) was utilized for assessment of key pathways between two groups (Fig. 4B). Among these enriched pathways, angiogenesis-related pathways including PI3K/Akt, JAK/STAT and Notch were selected (Supplementary Table 1). GSCs were treated with scrambled shRNA targeting IFITM3, and key protein expressions of these pathways were determined. Immunoblotting assay indicated that IFITM3 in GSCs regulate key protein expression in JAK/STAT3 signaling pathway (Fig. 4C). Next, GSCs were transfected with lentiviral vector encoding IFITM3, and co-incubated with endothelial cells. In vitro angiogenic assays displayed enhanced tube formation and sprouting capacities in hBMECs co-cultured with IFITM3-GSCs (Fig. 4D). Moreover, when WP1066 (JAK inhibitor) was added into GSCs culture medium, IFITM3-induced angiogenesis was significantly attenuated (Fig. 4D). In human GBM specimen, we observed elevated p-STAT expression in robust IFITM3-staining region (Fig. 4E). When fractionated IFITM3^+^ or IFITM3^-^ GSCs were injected orthopically into nude mice, IFITM3 expression was also consistent with p-STAT3 expression (Fig. 4F).

### GSC-derived IFITM3 contributes to angiogenesis via producing bFGF

**Fig. 5:**
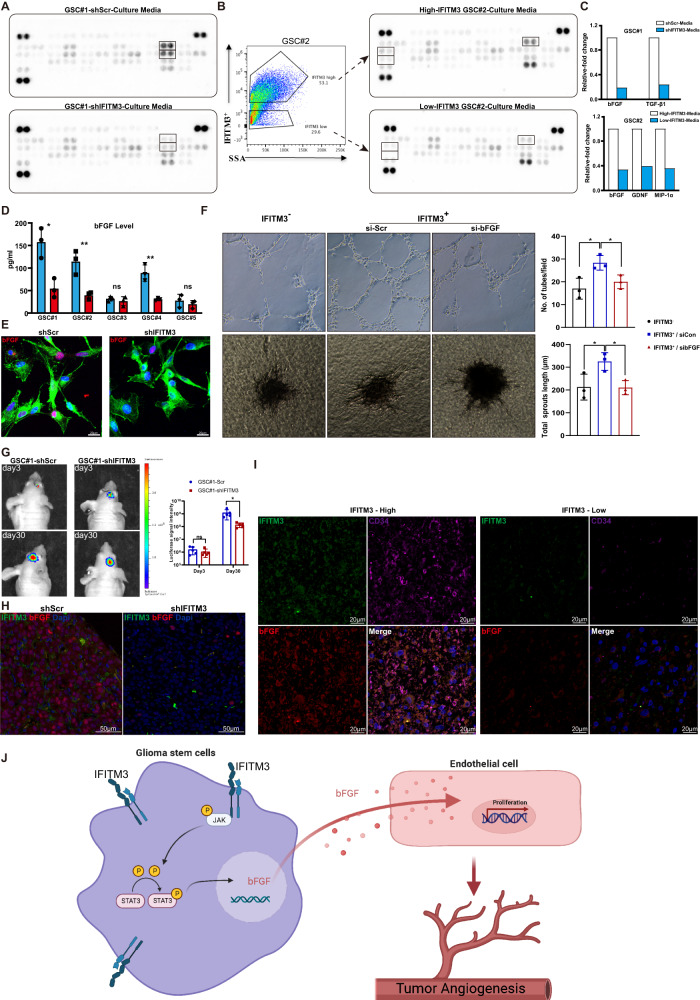
IFITM3 regulates GSC-mediated angiogenesis process in a bFGF-dependent manner. **A** GSC#1-shScr or GSC#1-shIFITM3 were cultivated for 24 h, of which culture media were harvested for cytokine detection using angiogenesis antibody array. **B** GSC#2 cells were fractionated into IFITM3+ or IFITM3-groups, and angiogenic cytokines were measured. **C** Bar charts show markedly altered cytokines of (**A**) and (**B**). **D** Secreted bFGF levels of GSC#1–5 were measured using Elisa assay. **E** Representative immunofluorescent images with bFGF (red) staining of GSC#1-shScr and GSC#1-shIFITM3. F-actin (green) is stained with phalloidin. Nuclei are counterstained with Dapi (blue). Scale bar = 50 μm. **F** Angiogenic abilities of fractionated IFITM3^−^ and IFITM3^+^ GSCs are measured, with the latter cells treated with manipulated bFGF downregulation. **G** Intracranial xenograft models are constructed with GSC#1-shScr and GSC#1-shIFITM3. Tumor sizes are monitored by in vivo imaging. **H** Representative immunofluorescence images staining for IFITM3 (green) and bFGF (red) and in GSC#1-shScr or GSC#1-shIFITM3 xenografts. **I** Representative immunofluorescence images staining for IFITM3 (green), bFGF (red), and CD34 (purple) in human GBM samples. **J** Schematic illustration of GSCs-mediated glioma angiogenesis through IFITM3-JAK/STAT-bFGF signaling pathway. Results are represented as Mean ± SD of biologically triplicate assays. *p < 0.05, **p < 0.01, ***p < 0.001. ns not significant.

The GO term molecular function (MF) analysis indicated that cytokine activity was significantly enriched in IFITM3-regulated genes (Supplementary Fig. 2). Secreted cytokines have powerful influence on angiogenesis either in normal tissue or malignant tissue. To identify the angiogenic factors involved in IFITM3-mediated angiogenesis, an angiogenesis array was performed. The expression of bFGF and TGFβ1 markedly decreased in shIFITM3 GSCs as compared with shCon-GSCs (Fig. 5A, C). When GSC#2 cells were fractionated into two groups with high or low IFITM3 expression. Culture media from high-IFITM3 cells showed increased bFGF level (Fig. 5B, C). In various GSCs, IFITM3-regulated bFGF production was further validated (Fig. 5D, E). Interestingly, a co-cultivation of GSCs with hBMECs revealed that blocking bFGF would substantially mitigate pro-angiogenic effect by IFITM3 (Fig. 5F). To explore IFITM3-mediated angiogenesis in vivo, we established intracranial mice model by implanting Luc labeled GSC#1 into nude mice. Tumor growth was monitored using in vivo Imaging System. Results indicated that IFITM3 knockdown exhibited attenuated tumor growth (Fig. 5G) and declined bFGF secretion (Fig. 5H). In human GBM samples, our data showed that IFITM3 expression was associated with bFGF production and enhanced angiogenesis (Fig. 5I). These findings indicated that GSC-derived IFITM3 contributed significantly to angiogenesis in vivo via regulating bFGF.

## Discussion

In the present study, we describe a novel mechanism of GSCs-induced GBM angiogenesis through expression of IFITM3 and bFGF (Fig. 5J). IFITM3 is a interferon-induced protein mediating antiviral activity via suppressing the entry of viruses [33]. Interestingly, it has been gradually unraveled that IFITM3 correlates with tumor progression, including proliferation [34], invasion [35], chemoresistance [36], metastasis [37], as well as angiogenesis [38]. A Recent finding indicated that TGFβ-regulated IFITM3 expression facilitates glioma cell invasion [39]. However, further investigation regarding molecular mechanism, by which IFITM3 influences glioma progression remains unclear. Conclusive evidence is now provided that IFITM3 functions as an angiogenesis inducer. Angiogenesis is the formation of new blood vessels including multiple components, such as the endothelial cell proliferation, cell migration, cell adherence and in vitro tube formation [40]. In the present research, we focused our investigations on GBM stem cell-induced alterations on endothelial cells. we utilize a co-cultivation system to demonstrate that IFITM3 is capable to stimulate endothelial cell proliferation, migration and sprouting through interaction between GSCs and endothelial cells. The observation that conditioned media from GSCs, as well as physically separated GSCs modulates human brain endothelial cell phenotypes indicates that GSCs release pro-angiogenic cytokines in a IFITM3-dependent method.

It has gradually become clear that cancer stem cells (CSCs) exist in various types of tumors tissues, even though the controversy about exact markers to identify and isolate CSCs [41]. Recent researches have unveiled that GSCs share similar characteristics. Both cell types express stem cell markers and the ability of self-renewal [42]. CD133 and Nestin have been widely considered as markers in GSCs [43]. However, there is also evidence that CD133-negative cells cultured from GBM patients harbored a self-renewal ability [44]. The present study demonstrates that IFITM3 is preferentially expressed in GSCs and decreased markedly in differentiated glioma cells, yet has no significant impact on GSC self-renewal ability (Fig. 3A). In fact, IFITM3 has been reported to exert no effect on cell proliferation and invasion in glioma cell lines [45], indicating that IFITM3 had no direct effect on glioma cells. These evidences suggest that IFITM3 might not be an indispensable factor in stemness maintenance of cancer cells, despite the close correlation between protein expression and stemness status. Furthermore, hypoxic situation led to a dramatic elevation of IFITM3 expression (Fig. 2G, H). Hypoxia is known to affect the CSCs maintenance and functions. Stem cells tend to residence in hypoxic regions within tumors, which probably benefit the preservation of tumor stemness [46]. And hypoxia could regulate a variety of targets related to tumor stemness and invasiveness. Our data is consistent with a prior study by Cai that high-IFITM3 expression correlated with high hypoxia score in the bladder cancer using bio-informatic method [47]. Rajan et al. demonstrated that gene expression of IFITM3 was elevated in microglia when rat brain suffered a transient cerebral ischemia [48]. Data from a study by Harmon and colleagues showed that ischemic injury in aged brains following stroke resulted in the induction of IFITM3 proteins [49]. Therefore, IFITM3 would act as a hypoxia-induced intermediate factor in GSCs, and subsequently the affects the tumor microenvironment surrounding GSCs.

Besides from tumor initiation, CSCs are shown to interact with endothelial cells and be closely linked to vasculature formation in perivascular niche [50]. Recent findings indicate that GSCs contributed to tumor vasculature through direct differentiation or releasing pro-angiogenic cytokines. GSCs cultured from GBM patients differentiated into vascular endothelial cells and pericytes when induced by serum in vitro [51, 52]. These GSCs-derived vascular cells harbored the tube formation capacity and could be identified in human GBM tissues [53]. In our study, IFITM3^high^ GSCs confers endothelial cells proliferation, migration and sprouting through paracrine.

Through human angiogenesis antibody array, we identified bFGF as a key motivator in GSC-mediated angiogenesis. High bFGF level has been reported in individuals with various categories of neoplasms and predicted a poor prognosis [54]. Elevated bFGF levels have been identified in human glioma, suggesting its importance in tumor growth progression [55]. Besides, one of the most prominent reason for the resistance to anti-VEGF treatment is the compensatory mechanism of other growth factors, with the bFGF being at the top of the list [54]. These results showed that IFITM3-bFGF axis between GSCs and endothelial cells might be the compensatory angiogenic approach when VEGF-dependent angiogenesis is interrupted.

In summary, the present study discloses a functional role IFITM3 in regulating GBM angiogenesis and identifying the downstream effectors, which are important for GBM progression. And bFGF is shown to be a downstream target of IFITM3 that modulates brain endothelial cell proliferation, migration and sprouting. Our findings establish the rationale for developing anti-vascular therapy based on the targeted disruption of IFITM3.

### Supplementary information

Supplementary figures and legends

Checklist

Supplementary Fig.1

Supplementary Fig.2

Supplementary Table S1

Supplementary Table S2

Supplementary Table S3

Original blots data

## Data Availability

All data generated in the present study are included either in the main article or in the supplementary information files.
