# Supplementary material for: IFITM3 promotes glioblastoma stem cell-mediated angiogenesis via regulating JAK/STAT3/bFGF signaling pathway
Source: Cell Death Dis. 2024 Jan 13;15(1):45. doi: 10.1038/s41419-023-06416-5 (PMC10787840; doi:10.1038/s41419-023-06416-5)
Supplement: Supplementary file 1 — Supplementary figures and legends [file 41419_2023_6416_MOESM1_ESM.docx]

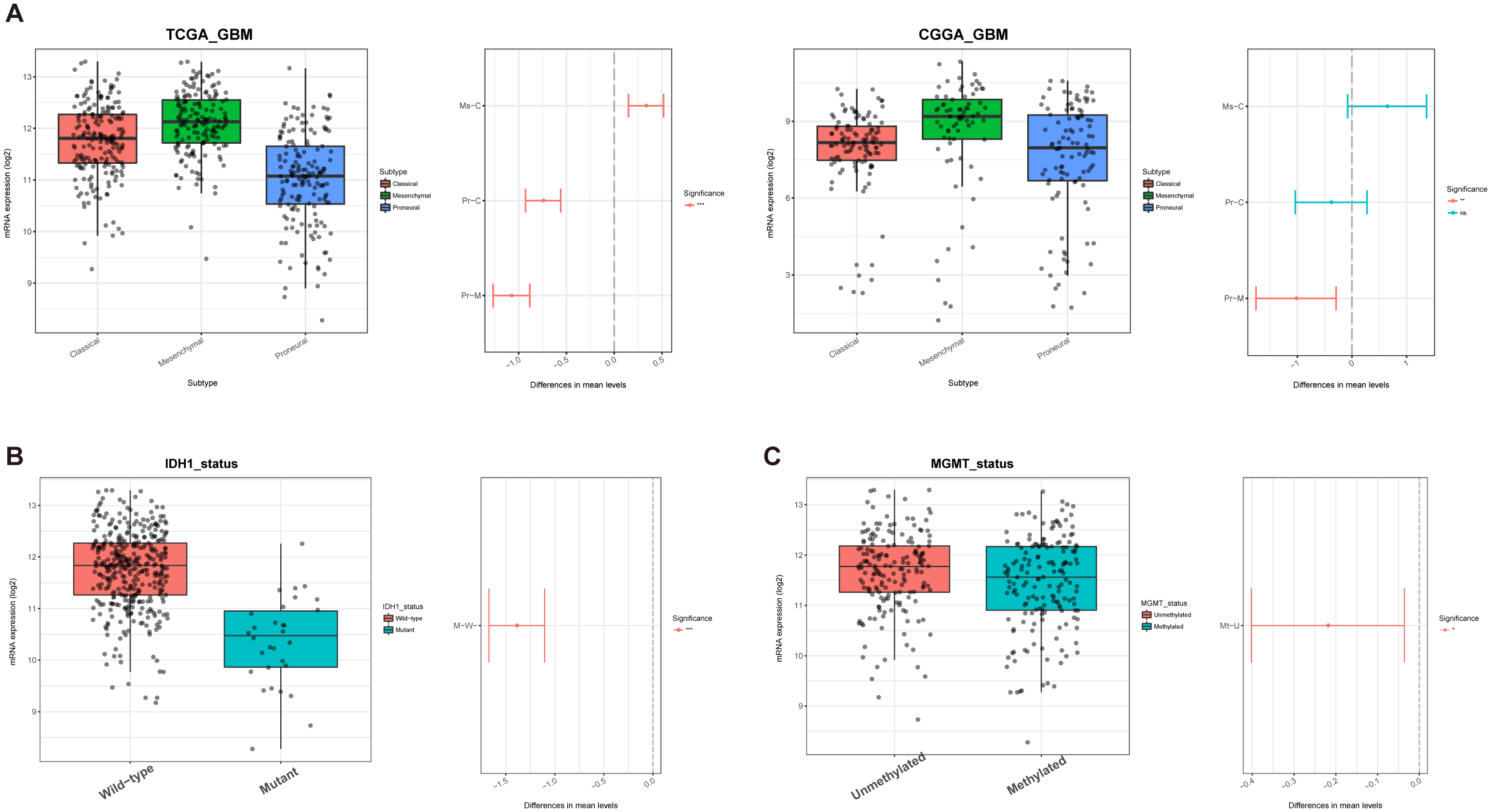


**Figure 1. Correlation between IFITM3 expression and prognostic factors of GBM patients.** (**a**) Association of IFITM3 with GBM subtypes in TCGA and CGGA GBM datasets. (**b**) Correlation between IFITM3 and IDH1 status in TCGA GBM dataset. (**c**) Correlation between IFITM3 and MGMT status in TCGA GBM dataset. **p< 0.05, **p< 0.01, ***p< 0.001. TCGA, The Cancer Genome Atlas; CGGA, Chinese Glioma Genome Atlas; MGMT, O6-methylguanine-DNA-methyltransferase.


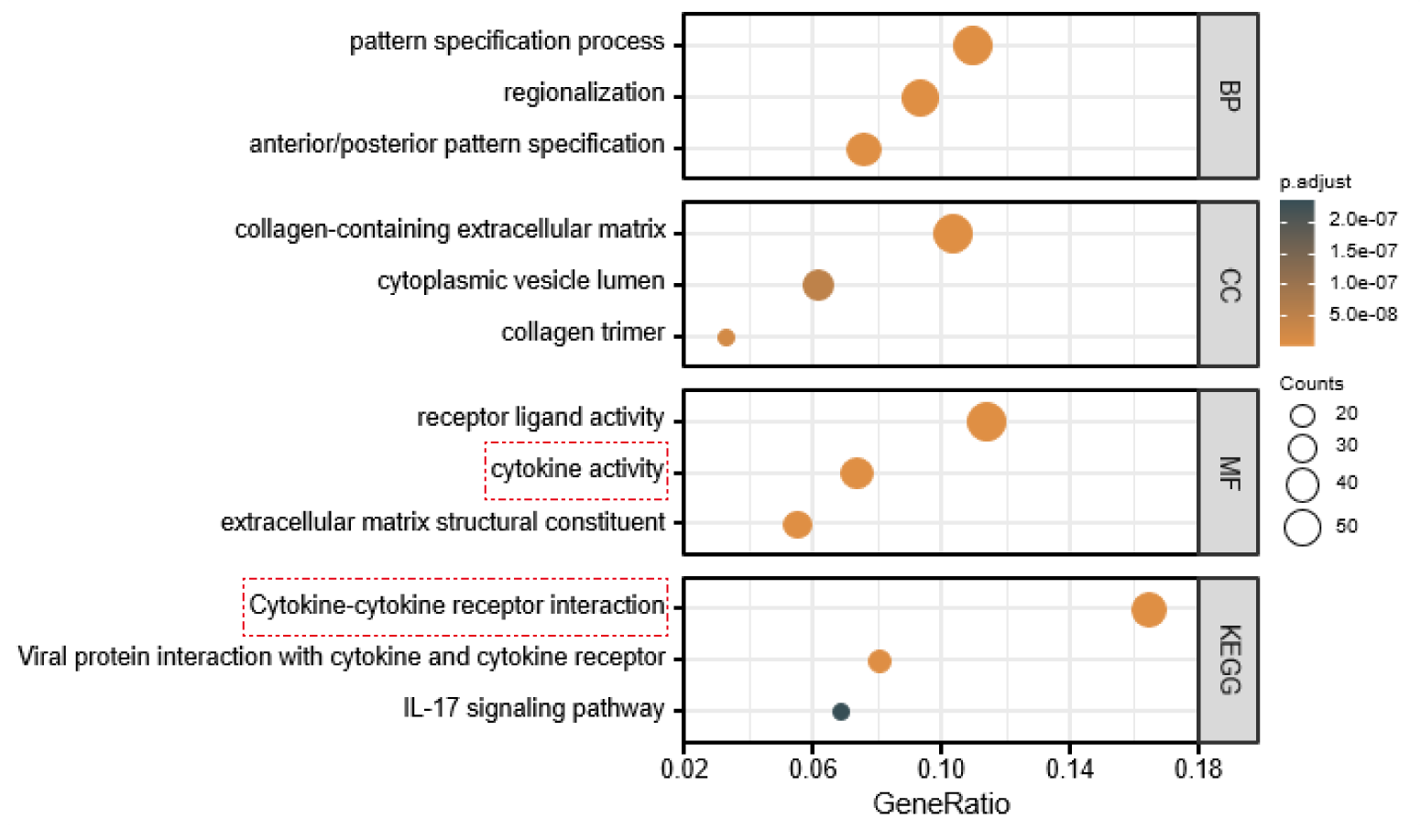


**Figure 2.** **Gene ontology (GO) and pathway enrichment analyses of differential expressed genes regulated by IFITM3.** Significantly enriched GO terms for BP, CC, MF and KEGG were analyzed using 1169 differential expressed genes. BP, biological processes, CC, cellular component, MF, molecular function.


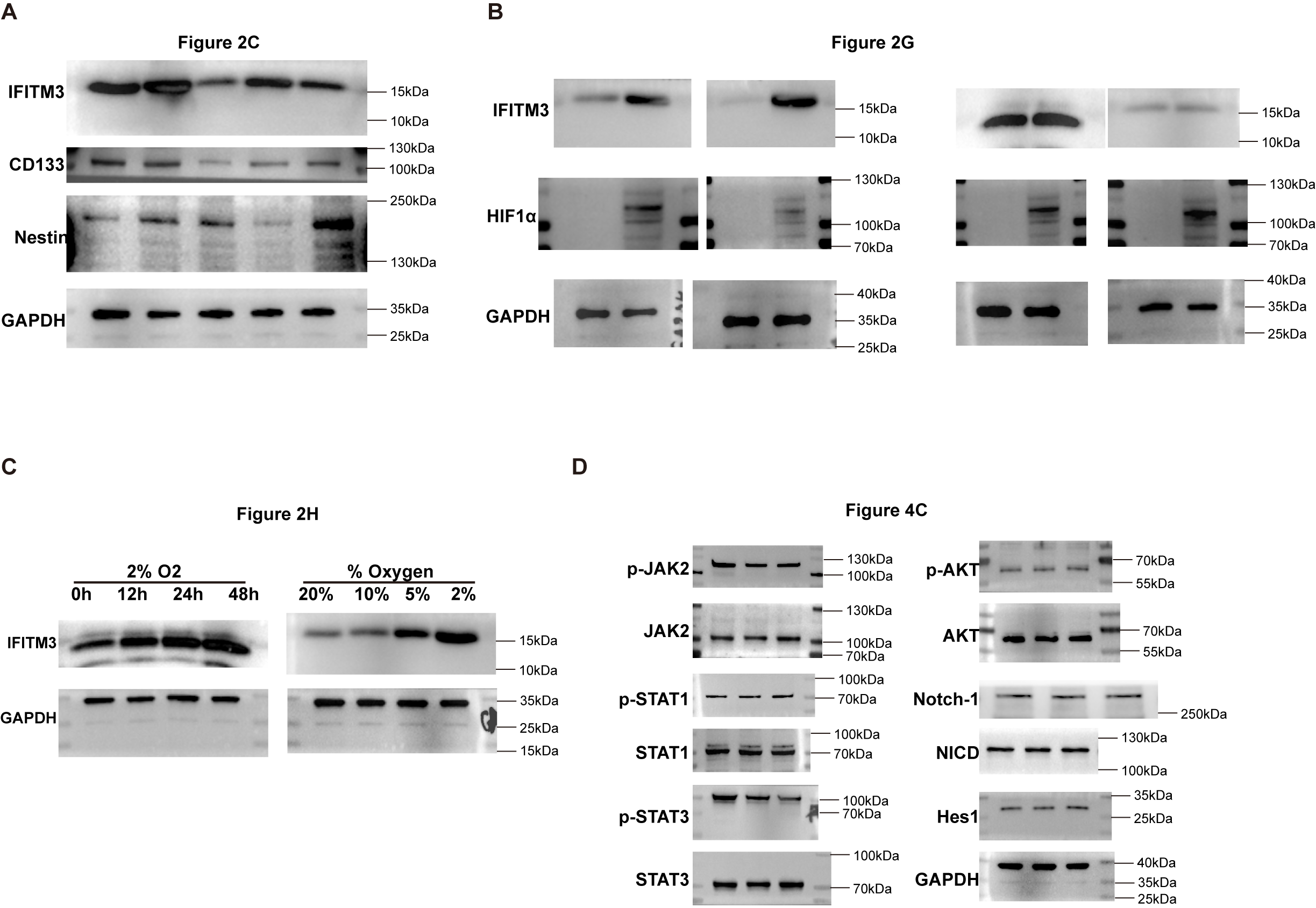


**Figure 3.** **Uncropped scans of the immunoblots**.
