# Supplementary figures and images for: IFITM3 promotes glioblastoma stem cell-mediated angiogenesis via regulating JAK/STAT3/bFGF signaling pathway
Source: Cell Death Dis. 2024 Jan 13;15(1):45. doi: 10.1038/s41419-023-06416-5 (PMC10787840; doi:10.1038/s41419-023-06416-5)

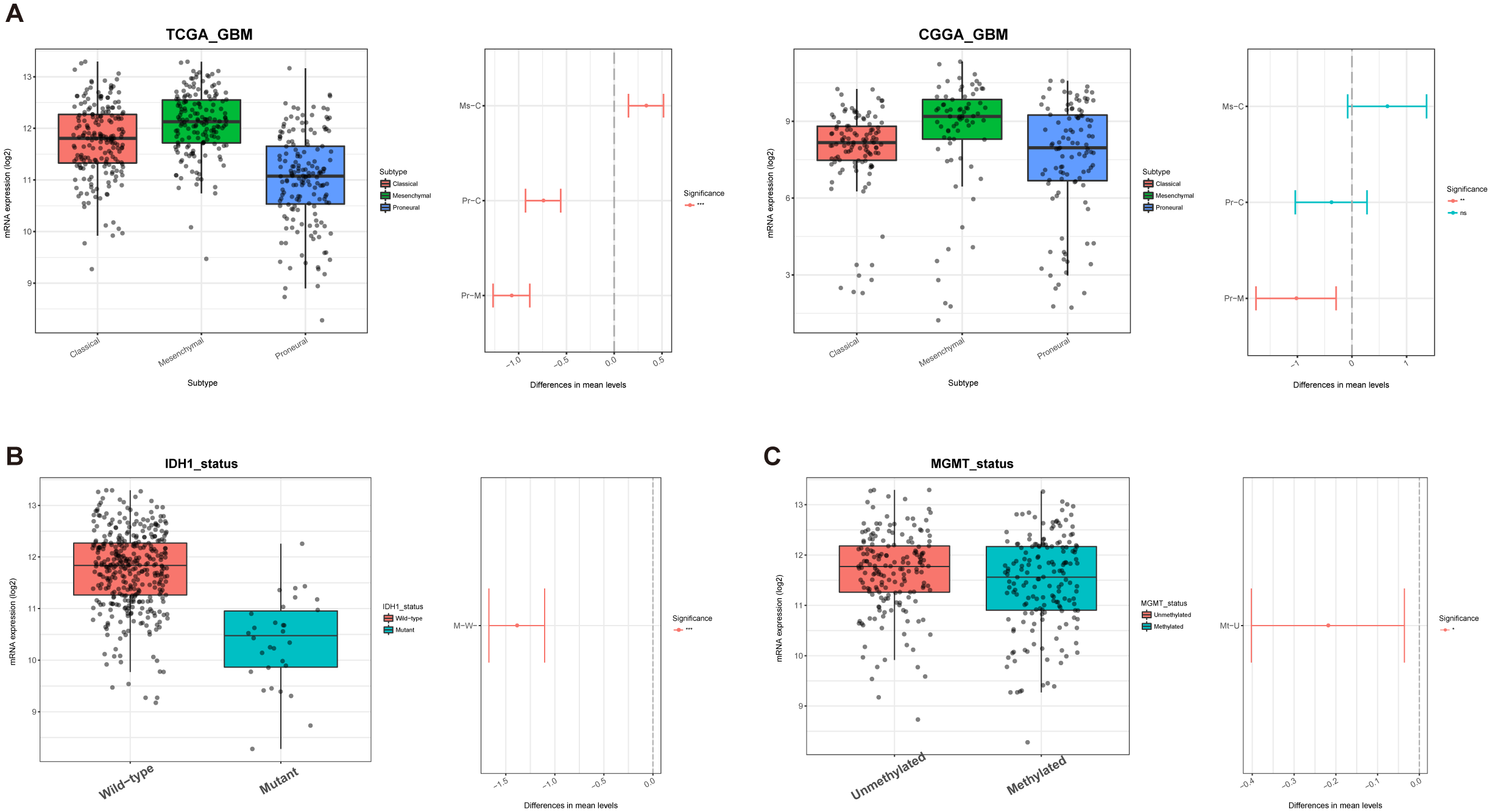

Supplement: Supplementary file 3 — Supplementary Fig.1 [file 41419_2023_6416_MOESM3_ESM.tif]

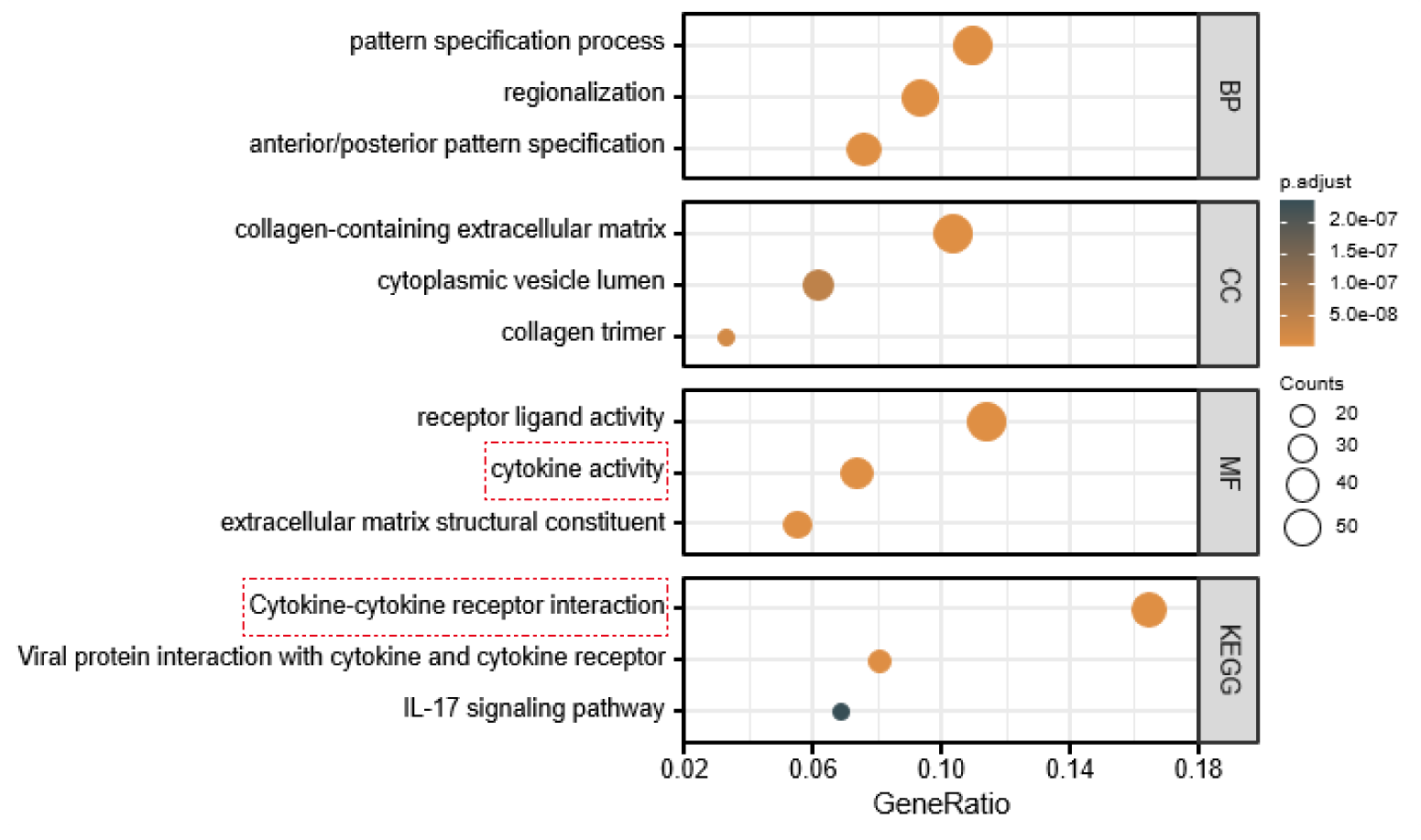

Supplement: Supplementary file 4 — Supplementary Fig.2 [file 41419_2023_6416_MOESM4_ESM.tif]

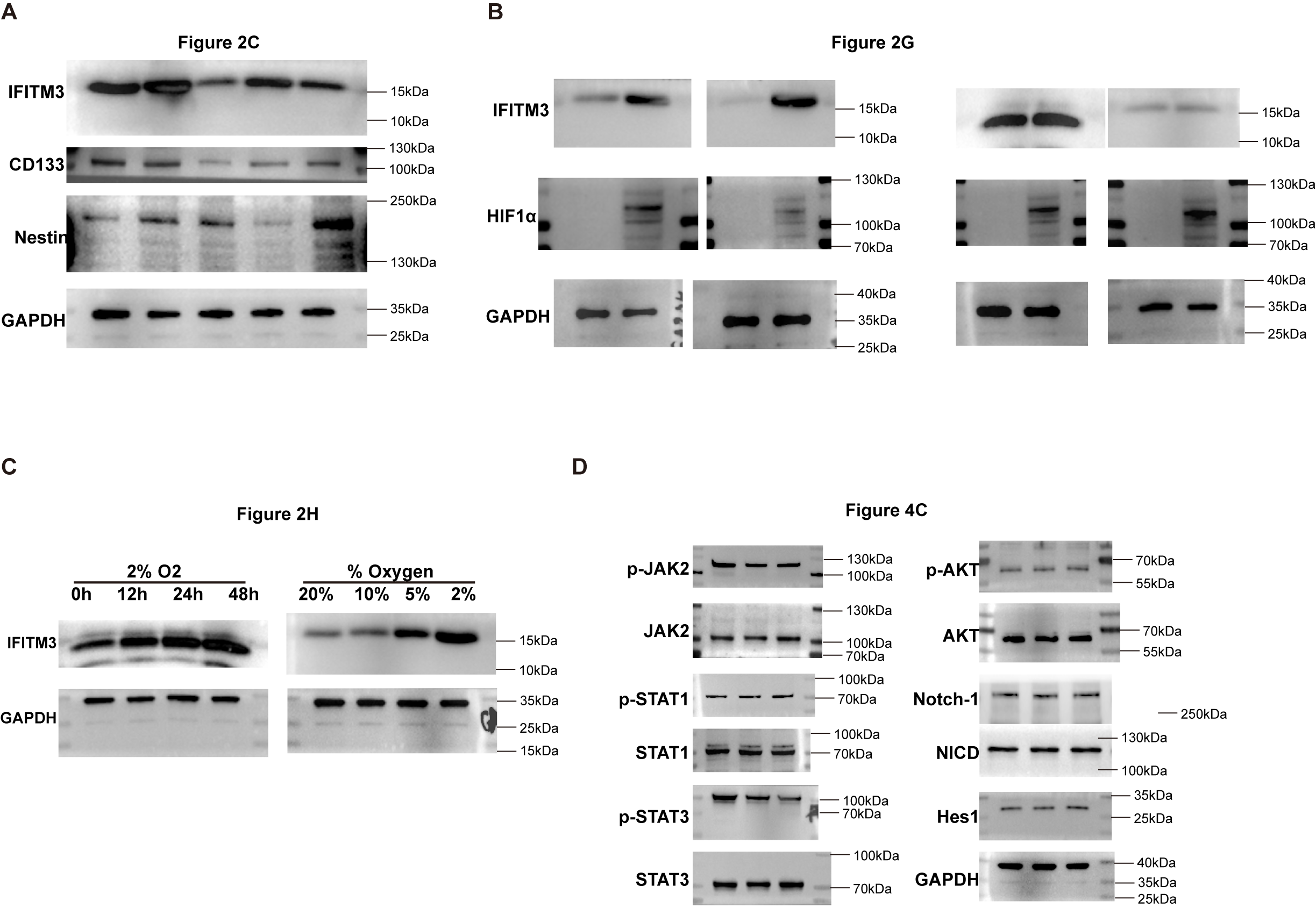

Supplement: Supplementary file 8 — Original blots data [file 41419_2023_6416_MOESM8_ESM.tif]
